# Public Health Response to Biological and Chemical Weapons: WHO Guidance

**DOI:** 10.3201/eid1101.040927

**Published:** 2005-01

**Authors:** James W. Buehler

**Affiliations:** * Emory University, Atlanta, Georgia, USA

**Keywords:** Public Health Response, Biological and Chemical Weapons, WHO Guidance, terrorist attack

In this manual, the World Health Organization (WHO) updates its guidance for governments in preparing for a possible terrorist attack with biological or chemical weapons. The book has something for virtually everyone who may have an interest in this topic, from government officials to clinicians, including information about the history of biological and chemical warfare, applicable international treaties, procedures for requesting WHO technical consultation, fundamentals of public health emergency response, basics of infectious disease treatment, treatment of patients with specific infectious or toxic exposures, physical properties of various agents, utility of reconnaissance satellites for detecting weapons development, management of food and water safety programs, etc.

And it is this ubiquity and ambitiousness that underlie the manual’s limitations and strengths. At times the guidance is so general that is almost an inventory of truisms (e.g., “If it is found that the [emergency] control measures are not effective, they must be changed or modified.”); elsewhere, the manual is a detailed resource. Its utility for different users will depend on their backgrounds and information needs. The core chapter, Public Health Preparedness and Response, may disappoint those seeking more than general principles. Yet these principles merit articulation as the foundation for prevention and response.

Descriptions of the sarin attack in Tokyo in 1995 and the anthrax attacks in the United States in 2001 illustrate lessons from governments’ recent experiences with chemical and biological terrorism. Both episodes demonstrate that relatively small attacks can have a profound impact and expose weaknesses in public health systems. The anthrax case study lauds the success of laboratory preparations but does not sufficiently address three essential questions: Why did clinicians caring for the initial patients with cutaneous anthrax not establish and report the diagnosis sooner? Why did the Centers for Disease Control and Prevention not recognize earlier that anthrax spores could escape from sealed letters? Why did the federal government stumble initially in its efforts to communicate with the public? For each question, an assessment of systemic hurdles would benefit readers seeking to improve the functioning of the public health system.

The manual generally, but not consistently, avoids bureaucratic lingo. While clearly organized, the book lacks an index, complicating efforts to find information quickly. The appendices on chemical and biological agents offer concise, formatted summaries similar to those available through other resources, but ironically provide relatively little information about the agents’ potential as weapons.

This manual will find a home on bookshelves worldwide among government officials and others concerned about the threat of biological and chemical terrorism. For those in countries most in need of this guidance, its scope may be overwhelming. But the book’s underlying theme—that public health preparedness for biological or chemical terrorism depends on fundamental capacities to respond to more common health threats—is its most salient message, no matter where the user resides.

